# A tactile and airflow motion sensor based on flexible double-layer magnetic cilia

**DOI:** 10.1038/s41378-022-00478-9

**Published:** 2023-01-17

**Authors:** Jiandong Man, Junjie Zhang, Guangyuan Chen, Ning Xue, Jiamin Chen

**Affiliations:** 1 State Key Laboratory of Transducer Technology, Aerospace Information Research Institute, Chinese Academy of Sciences, 100190 Beijing, People’s Republic of China; 2 School of Electronic, Electrical and Communication Engineering, University of Chinese Academy of Sciences, 100049 Beijing, People’s Republic of China

**Keywords:** Electrical and electronic engineering, Nanoparticles

## Abstract

Inspired by the concept of bionics, a tactile and airflow motion sensor based on flexible double-layer magnetic cilia is developed, showing extremely high sensitivity in both force and airflow detection. The upper layer of the magnetic cilia is a flexible material mixed with magnetic particles, while the lower layer is a pure flexible material. This double-layer structure significantly improves magnetism while maintaining cilia flexibility. In addition, a metal tube pressing (MTP) method is proposed to overcome the difficulties in preparing large aspect ratio (over 30:1) cilia, offering simplicity and avoiding the use of large-scale MEMS instruments. The developed sensor has a detection range between 0 and 60 µN with a resolution of 2.1 µN for micro forces. It also shows great detection ability for airflow velocity with a sensitivity of 1.43 µT/(m/s). Experiments show that the sensor could be applied in surface roughness characterization and sleep apnea monitoring.

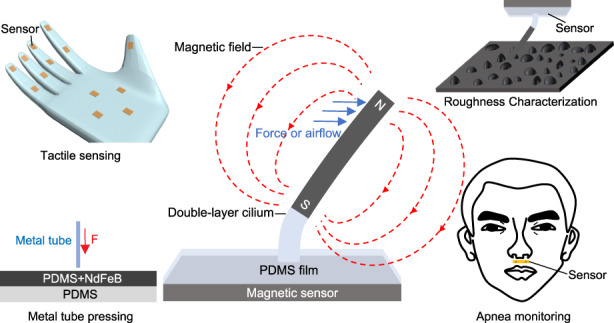

## Introduction

Tactile sensors help robots obtain a variety of tactile information, such as pressure, vibration, roughness, temperature and even airflow. Currently, tactile sensors have been widely used in various fields, such as intelligent manipulators^1,2^, minimally invasive surgery^3^, electronic skin^1,4^, intelligent prostheses^5^, and human–computer interactions^6^. Tactile sensors based on the principles of resistance^7^, capacitance^8,9^, and piezoelectrics^10^ have been developed. However, many issues and challenges remain to be overcome: (1) The detection limit of most reported tactile sensors for micro forces is normally over 1 mN, which is not sensitive enough. There is an increasing demand for tactile sensors with a lower detection limit in special situations such as surface roughness characterization^11^. (2) Generalized tactile sensing requires the perception of not only force but also airflow. For example, people can know whether there is wind just by stretching out their hands. However, this is difficult for robots with only tactile sensors. To measure airflow speed, it is necessary to install an additional sensor for robots. Therefore, enhancing the detection ability of tactile sensors to perceive micro forces and even airflow is significant for the intelligence and miniaturization of robots.

The ciliary structure is one of the most sensitive structures known in nature, whether the lateral line of fish, the hair on spiders’ legs or the antennae of mosquitoes. A ciliary structure helps organisms realize sensitive perception of the environment. The application of ciliary structures can greatly improve the detection ability of tactile sensors^12–14^. With the rapid development of magnetic sensors, it is possible to detect small changes in a magnetic field during the bending of magnetic cilia^15,16^. To produce magnetic cilia, some studies have used the demolding method^17–25^. A hard mold is perforated first by laser processing or etching with photolithography. Then, a mixture of a flexible matrix and magnetic particles is poured into holes in the mold. After being solidified, the mixture is peeled off from the mold to form cilia. According to reported results, the detection limit for micro forces can reach as low as 31 µN^19^. However, there are still many problems to be solved. First, to enhance the magnetism of cilia, it is necessary to increase the mass ratio of magnetic particles as much as possible. Cilia with a magnetic particle mass ratio of 65% have already been used^24^. However, a further increase in magnetic particle content can severely reduce the flexibility of the cilia, which means that the magnetism and flexibility of cilia cannot be improved simultaneously. Second, as the mixture solidifies in the mold, it adheres closely to the inner wall. When the aspect ratio of cilia is large, the cilia break easily in the mold. In other words, the demolding method is not suitable for making large aspect ratio cilia. The aspect ratio of cilia in previous studies was 5:1 or even lower. Third, the demolding method requires large-scale equipment, such as laser cutters, etching instruments and lithography machines, which are costly and complex. Finally, it is difficult to control the distribution of magnetic particles flexibly.

To overcome the issues mentioned above, a tactile airflow sensor based on double-layer magnetic cilia is proposed in this study. The upper layer of the cilia contains a large amount of magnetic particles. The mass ratio of magnetic particles to the flexible matrix can reach 70%, which provides sufficient magnetism for cilia. The lower layer of the cilia is a pure flexible matrix, which ensures high flexibility. Therefore, this structure can achieve a balance of magnetism and flexibility. To address the inability of the demolding method to produce large aspect ratio cilia, a metal tube pressing (MTP) method is proposed. The aspect ratio of the cilia prepared by this method can exceed 30:1. In addition, the MTP method does not require large-scale MEMS equipment, so it is simple in process and low in cost. Benefiting from the significant performance of cilia, we combine cilia with a magnetic sensor to fabricate a tactile sensor. This new type of tactile sensor is extremely sensitive to micro forces and airflows and has shown excellent performance in surface roughness characterization and sleep apnea monitoring.

## Materials and methods

### Sensing concept

**Fig. 1:**
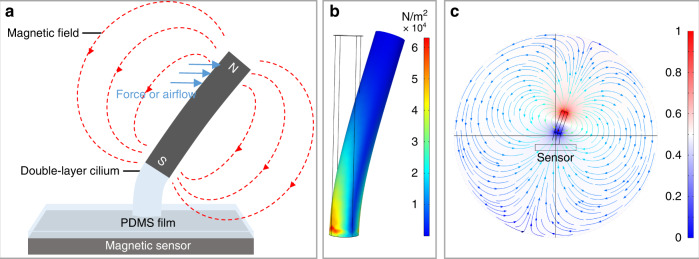
Illustration of the sensor and the simulation results of the magnetic cilium. **a** Illustration of the tactile and airflow sensor with a double-layer magnetic cilium. **b** Stress distribution of the cilium. **c** Distribution of the stray field around the cilium

The sensitive structure of a sensor can be an array of multiple cilia or a single cilium. For simplicity, a single cilium is used for description below. The cilium we use is a double-layer magnetic cilium based on polydimethylsiloxane (PDMS). The magnetism comes from the NdFeB (NdFeB is a permanent magnet material with high residual magnetization) particles mixed in the upper layer. The cilium is magnetized in a vertical upward magnetic field. When the external force acts on the cilium, the magnetic stray field around the cilium changes, as shown in Fig. 1a. The change in the stray field is detected by a magnetic sensor under the cilium. The magnitude and direction of the external force can be calculated according to the output of the magnetic sensor.

Theoretically, the cilium can be simplified as a vertical cylindrical cantilever beam fixed at the bottom. The relationship between the force and the deflection distance is shown in Eq. 1:

1 $$\delta = F\frac{{64l^3}}{{3\pi ED^{{{\mathrm{4}}}}}}$$

where *δ* is the deflection distance of the top of the cylinder; *F* is the force acting on the top of the cylinder; *l* is the length of the cylinder; *E* is the Young’s modulus of the material; and *D* is the diameter of the cylinder.

Under the same force, the larger the deflection distance of the cilium tip is, the greater the variation in the stray magnetic field, and the higher the sensitivity of the sensor. It can be seen from the equation that the deflection distance of the cilium tip can be improved from two aspects: reducing the Young’s modulus and increasing the aspect ratio (increasing the length and reducing the diameter) of the cilium. We optimize the cilium through these two aspects exactly. First, the Young’s modulus of the whole sensitive structure is reduced by using pure PDMS at the bottom of the cilium. Second, the aspect ratio is increased by using the MTP method.

The magnetic sensor is a linear Hall sensor (Melexis, MLX90393). When the external magnetic strength changes, the Hall material in the sensor generates a Hall voltage. The size of the Hall voltage is shown in Eq. 2:

2 $$V_H = \frac{{I_SB}}{{ned}}$$

where *n* and *d* are the carrier concentration and thickness of the Hall material, respectively, which are only related to the Hall material itself. *e* is the electronic charge, which is a fixed value. *I*_*s*_ is the current flowing through the Hall material, and *B* is the external magnetic strength. When the current *I*_*s*_ is fixed, the Hall voltage *V*_*H*_ is only positively related to the change in magnetic strength. Therefore, the external force or airflow can be obtained by collecting and processing *V*_*H*_.

### Simulation

COMSOL Multiphysics is used to simulate the distribution of the stress and magnetic stray field. First, we simulate the stress distribution by a solid mechanics component. A fixed constraint is added to the bottom of the cilium, and a transverse force is applied to the top. As shown in Fig. 1b, the stress is mainly distributed at the bottom of the cilium. Therefore, replacing this part with pure PDMS (low Young’s modulus) can improve the flexibility of the cilium. We also simulate the distribution of the stray field around the cilium by setting the magnetic scalar potential at both ends of the cilium. The result is shown in Fig. 1c. The stray field is tilted when the cilium is bent. The direction of the stray field at the magnetic sensor under the cilium changes from vertical to oblique.

### Fabrication

**Fig. 2:**
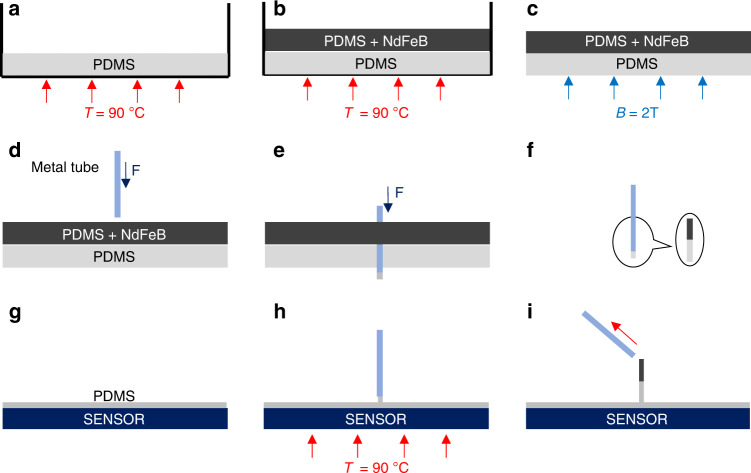
Fabrication process of the tactile sensor by using the MTP method. **a** PDMS solvent and curing agent are mixed and heated in a Petri dish. **b** PDMS and NdFeB magnetic particles are mixed and then solidified above the pure PDMS. **c** The double-layer film is magnetized in a strong magnetic field. **d** A metal tube is used to make the double-layer cilium. **e** The metal tube is passed vertically through the double-layer film. **f** A double-layer cilium remains in the metal tube. **g** A layer of uncured pure PDMS is placed on the surface of the magnetic sensor. **h** The lower end of the metal tube contacts with the PDMS on the sensor surface. **i** The double-layer cilium is separated from the metal tube

The fabrication process of the tactile sensor using the MTP method is shown in Fig. 2.

First, PDMS (Sylgard 184 Silicone Elastomer, Dow Corning Corporation) solvent and curing agent are mixed in Petri dish 1 and put in a vacuum oven for 30 min to remove bubbles. Next, the dish is placed in a baked oven at 90 °C for 20 min to solidify the pure PDMS initially, as shown in Fig. 2a. Second, PDMS solvent and curing agent are mixed in the same ratio in Petri dish 2, and then a certain mass of NdFeB magnetic particles with an average diameter of 5 µm is added. The mass can be adjusted flexibly according to needs. After being fully mixed, the mixture is poured into Petri dish 1. The thickness of the mixture can also be adjusted flexibly. Similarly, dish 1 is placed in a vacuum oven for 30 min. Then, it is placed in a baked oven at 90 °C for 1 h to solidify completely, as shown in Fig. 2b. It is noted that PDMS with different flexibility can be obtained by using different curing temperatures (as shown in Fig. S1). Third, the solidified double-layer film is magnetized in a strong magnetic field (magnitude: 2 T; direction: vertical upward), as shown in Fig. 2c. Fourth, a metal tube (such as a flat syringe needle, dispensing machine needle or custom stainless steel tube) with an inner diameter of 120 µm is used. It is passed vertically through the double-layer film from top to bottom. After passing through, a double-layer cilium remains in the metal tube. At the moment of penetration, the flexible body squeezed at the end of the metal tube is released, so a part of the flexible body is extruded out of the metal tube (this is for removing the cilium from the tube), as shown in Fig. 2d–f. Fifth, a layer of uncured pure PDMS is placed on the surface of the magnetic sensor. The sensor is adhered to a lifting heating platform. The metal tube with the cilium is fixed above the platform. Sixth, the heating platform is raised slowly to make the cilium contact the PDMS on the sensor. Then, the sensor is heated at 90 °C for 1 h to solidify the pure PDMS completely, as shown in Fig. 2h. The inner wall of the metal tube is extremely smooth, so the sensor can be made by slowly pulling the double-layer cilium out of the metal tube, as shown in Fig. 2i.

**Fig. 3:**
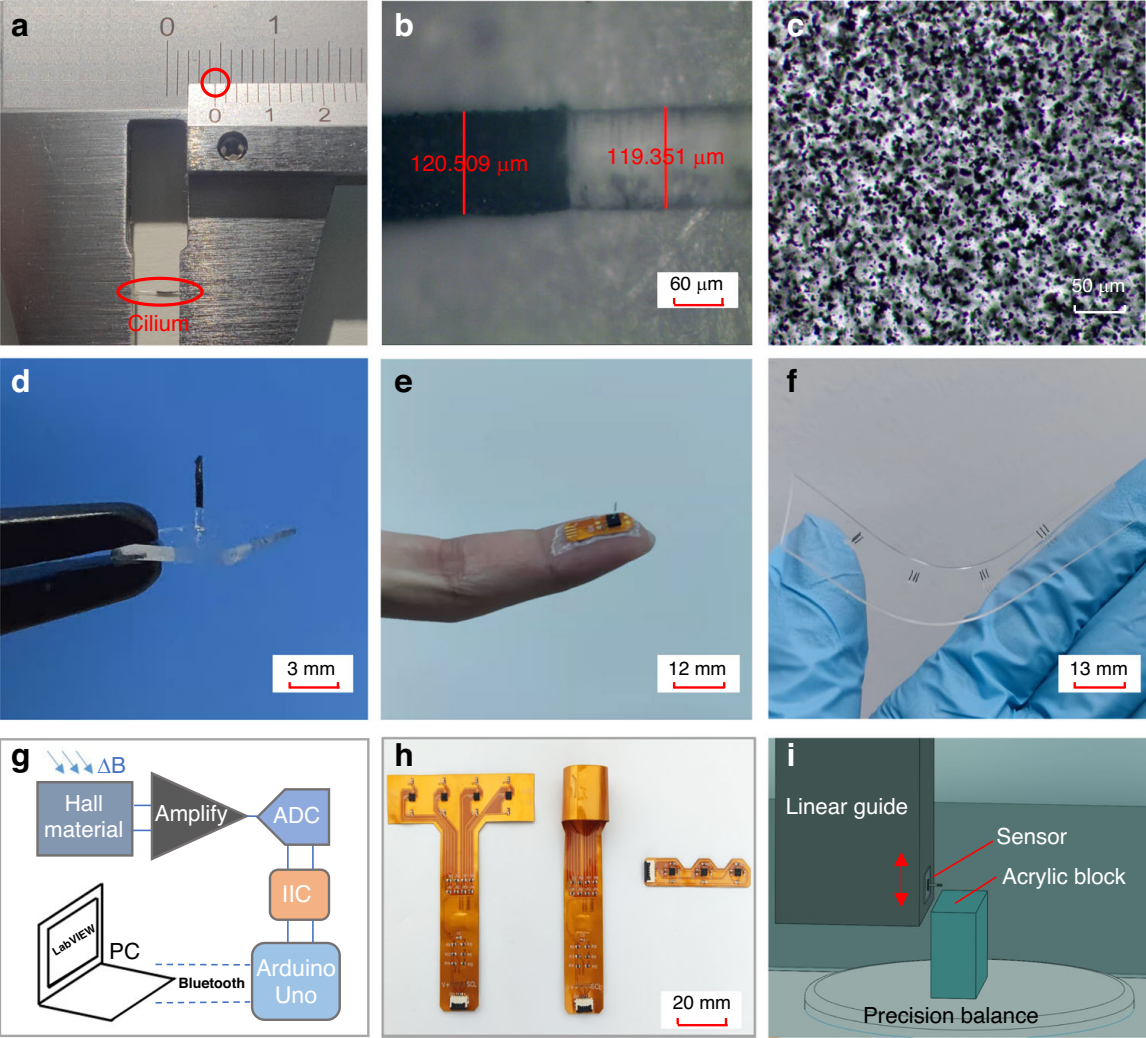
Photographs of the magnetic cilia sensor, FPC board and illustration of the testing system. **a** The length of the cilium measured by a Vernier caliper. **b** The diameter of the cilium. **c** SEM image of magnetic particles in the cilium. **d** Combination of a double-layer cilium and a PDMS film. **e** Photograph of the tactile sensor attached to a fingertip. **f** Photograph of multiple cilia arrays on a PDMS film. **g** Circuit diagram of this study. **h** Photograph of FPC boards. **i** Illustration of the precision testing system

The length of one cilium produced by the MTP method is shown in Fig. 3a, which is more than 4 mm. Figure 3b shows the diameter of the cilium at the boundary of different layers. It can be seen that the diameter is approximately 120 µm. Therefore, the aspect ratio of the cilium is above 30:1. Figure 3c shows an SEM (scanning electron microscope) image of magnetic particles in the cilium. The light part is the PDMS flexible body, and the dark and reflective parts are NdFeB particles. Figure 3d shows the combination of a double-layer cilium and a pure PDMS film, which is removed from the surface of the magnetic sensor. Figure 3e shows a photograph of attaching a tactile sensor to a fingertip. Depending on the application requirements, the MTP method can be further extended to fabricate cilia arrays. First, several metal tubes are combined to form a tube array. Then, the tube array can be used to fabricate cilia arrays. Figure 3f shows a photograph of multiple cilia arrays that can be used in airflow velocity measurement.

The circuit diagram of this study is shown in Fig. 3g. The Hall voltage is first amplified by an operational amplifier and then converted to a digital signal by an ADC (analog-to-digital conversion) module. The digital signal is sent to the Arduino Uno controller through the IIC (inter-integrated circuit) protocol, and then it is sent to a PC through Bluetooth. Several flexible printed circuit (FPC) boards are designed to meet the demands of practical applications. The FPC board for airflow velocity measurement is shown on the left in Fig. 3h. It can be equipped with four magnetic sensors: three working sensors and one reference sensor. The cilia arrays in Fig. 3f can be attached to the surface of the magnetic sensors. The FPC board is then rolled up to form a pipe for the passing of airflow, as shown on the middle in Fig. 3h. An FPC board for sleep apnea monitoring is shown on the right in Fig. 3h. It can be equipped with three magnetic sensors: two for working and one for reference. According to the shape of human nostrils, the FPC board is designed with two protrusions. The protrusions can be placed closer to the nostrils for respiratory monitoring.

### Characterization

The Young’s modulus and magnetic properties of PDMS with magnetic particles were tested with a push-pull tester (MARK-10, ESM303) and a physical property measurement system (Quantum Design, PPMS with VSM-9T). An airflow waveform generator (Piston, PWG-33BT) was used to characterize the response to airflow velocity. To characterize the response to micro forces, we established a precision testing platform. The platform consists of a linear guide (FUYU, FSL40XYZ-L) and a 1/10,000 g precision balance (Sartorius, BSA124S), as shown in Fig. 3i. The sensor was installed at the side of the lower end of the guide. An acrylic block was placed on the balance so that the top of the cilium was on the edge of the acrylic block. When the guide moved vertically down, the cilium touched the acrylic block and bent. The sensor, the balance and the guide could communicate with a PC through an RS232 serial port, and LabVIEW software was used to read, display and save data simultaneously.

## Results and discussion

### Basic performance of the sensor

**Fig. 4:**
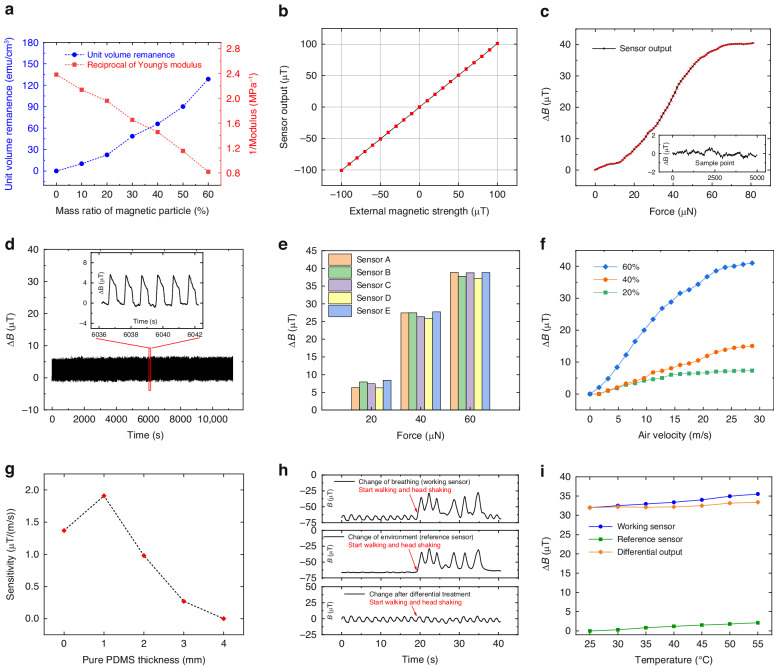
Basic performance of the sensor. **a** Effect of magnetic particle content on the magnetism and flexibility of PDMS. **b** Magnetic properties of MLX90393 under a weak magnetic field. **c** Response of the sensor to micro forces. Inset: noise of the sensor when there is no input. **d** Durability test results of a cilium with more than 10,000 bends. Inset: enlarged view of the signal waveform. **e** Reproducibility of this sensor. Five sensors with labels of A, B, C, D and E were produced independently. **f** Response of the sensor to airflow velocity. **g** Influence of the thickness of the pure PDMS layer on airflow sensitivity. **h** Results of using a reference sensor to reduce the influence of the external magnetic field. **i** Temperature stability of the airflow sensor

To characterize the effect of magnetic particle content on the magnetism and flexibility of PDMS, we fabricated PDMS films (length: 6 mm; width: 5 mm; thickness: 1 mm) with different magnetic particle contents. The test results of the unit volume remanence and Young’s modulus are shown in Fig. 4a. With increasing magnetic particle content, the unit volume remanence of the films increased, which was beneficial for improving the performance of the sensor. However, the flexibility decreased (the right ordinate in Fig. 4a is the reciprocal of Young’s modulus). Therefore, the double-layer structure was used to obtain magnetism and flexibility simultaneously in this work.

The maximum working range of MLX90393 can reach 5 mT. To characterize its magnetic properties under a weak magnetic field, we performed a performance test experiment. A magnetic shielding barrel and a Helmholtz coil were used to test the output of the sensor in the magnetic range of –100 to 100 µT. This range is also the working range used in all subsequent experiments of this study. The results are shown in Fig. 4b. The vertical axis is the magnetic strength acquired and converted with LabVIEW. The linearity of this sensor is excellent, with a value of 1.15%.

To characterize the response of the sensor to micro forces, we tested a single cilium with a magnetic particle mass ratio of 70%. The results are shown in Fig. 4c. Δ*B* is the difference between the magnetic strength after and before the bending of the cilia. The linear operating range of the sensor is 0–60 µN. In the linear operating range, the sensitivity of the sensor is 0.63 µT/µN. The inset of Fig. 4c shows the noise of the sensor when there is no input. The noise range of the sensor is 1.3 µT. According to the sensitivity, the resolution of the sensor is calculated to be 2.1 µN. Fig. S2 shows the change in the output signal when a feather (18 mg) is placed and then removed slowly on the sensor (only one working sensor on the FPC board was used). The output signal obviously changes, which indicates that the sensor can be applied to the manipulator to grab light and fragile objects.

Durability should be considered when a flexible material is used as a sensing structure. The magnetism of the cilium also needs to be taken into account during bending. We performed a repetitive bending experiment over 10,000 times (at 1 Hz) to characterize the durability and magnetism. The results are shown in Fig. 4d. As the number of tests increases, there is no obvious drift in the output of the sensor, which indicates that the sensor has great repeatability and durability. In addition, to demonstrate the reproducibility of this sensor, we produced five sensors with labels of A, B, C, D, and E independently. We precisely controlled the length, thickness and magnetic particle content of different cilia. Then, small forces of 20, 40, and 60 µN were applied to the five sensors. The outputs are shown in Fig. 4e. Although there are some differences, the trend of the sensor output increasing with increasing force is similar. In future mass manufacturing, the reproducibility of this sensor could be further improved by using more professional batch equipment.

To obtain the response of the sensor to airflow velocity, the FPC board mentioned above was rolled up to form a pipe. An airflow waveform generator was used to generate airflow with different velocities. Three cilia with magnetic particle contents of 20%, 40%, and 60% were tested. The results are shown in Fig. 4f. The outputs of the sensors with different cilia are all proportional to the airflow velocity. Moreover, with the increase in the content of magnetic particles, the sensitivity of the sensor increases. The sensitivity of the sensor with a magnetic particle content of 60% is calculated to be 1.43 µT/(m/s). This sensitivity can be further enhanced by forming cilia into an array or reducing the overall size of the cilia. These results indicate that this sensor can serve as a new type of device to sense airflows.

To explore the influence of the thickness of the pure PDMS layer on airflow sensitivity, we used five cilia with a total length of 4 mm (the length of their pure PDMS layer is 0, 1, 2, 3, and 4 mm) for the test. As shown in Fig. 4g, the airflow sensitivity first increases and then decreases with increasing length of the pure PDMS layer. This is because the airflow sensitivity is affected by two factors: the flexibility and magnetism of cilia. When pure PDMS is used to replace the PDMS containing a large amount of magnetic particles below, it is easier to bend under airflow because of its low Young’s modulus. However, with the increase in the length of the pure PDMS layer, the total content of magnetic particles in cilia decreases. As a result, the change in the magnetic strength at the magnetic sensor decreases, which leads to a decrease in the sensitivity.

The interference of an external magnetic field must be considered when magnetic sensors are used. In this study, a reference magnetic sensor was used to reduce the interference. To verify the effect, we used a working sensor and a reference sensor to detect the respiratory signals of a volunteer for ~40 s. The reference sensor detected only the change in the external magnetic field but not the change caused by the cilium. For the first 20 s, the volunteer sat still. In the last 20 s, the volunteer ran and shook his head significantly. The upper figure in Fig. 4h represents the output of the working sensor. After 20 s, the output changes dramatically, and the respiratory waveform cannot be distinguished. The middle figure in Fig. 4h shows the output of the reference sensor, and the lower figure in Fig. 4h shows the differential signal between the working sensor and the reference sensor. The difference restores the cluttered waveform to a state where respiratory peaks and valleys can be identified clearly. It is shown that the reference sensor is effective in eliminating the interference of the external magnetic field. By using the reference sensor, the tactile sensor can work in more complex situations, such as walking, running and cycling. Therefore, this tactile sensor has great application potential in wearable electronics.

Based on a similar method of using the reference sensor, the temperature drift of the sensor can also be reduced. We placed the sensor on a heat plate. Under the same airflow, the output of the sensor at different temperatures is shown in Fig. 4i. There are two reasons for the output change: the change in the Young’s modulus of the cilium and the temperature drift of MLX90393. The influence of the MLX90393 temperature drift can also be reduced by using the reference sensor. To illustrate the effect, we used two airflow sensors, and only one working sensor was in the airflow. The temperatures at these two sensors are the same. The differential output is shown in Fig. 4i. It can be seen that the temperature stability has been significantly improved after adjusting with the differential output.

A comparison of our work with other related works is shown in Table 1. It can be seen that the best advantage of our sensor is the powerful ability to detect micro forces. Among all magnetic tactile sensors, our sensor has the best resolution of micro forces, at 2.1 µN. Another key advantage of our sensor is that it can sense both tactile and airflow motions, which is a capability that most tactile sensors do not have.

**Table 1:** Comparison of different magnetic tactile sensors

Ref.	Resolution	Sensitivity	Airflow
^28^	10 mN	78 µV/mN	No
^29^	710 µN	8.5–29.8 µT/mN	No
^30^	-	0.08 µT/mN	No
^19^	31 µN	1.6 Ω/mN	Yes
^22^	333 µN	0.1 mV/mN	No
This work	2.1 µN	630 µT/mN	Yes

“-” indicates that there is no relevant information in the paper

### Surface roughness characterization

**Fig. 5:**
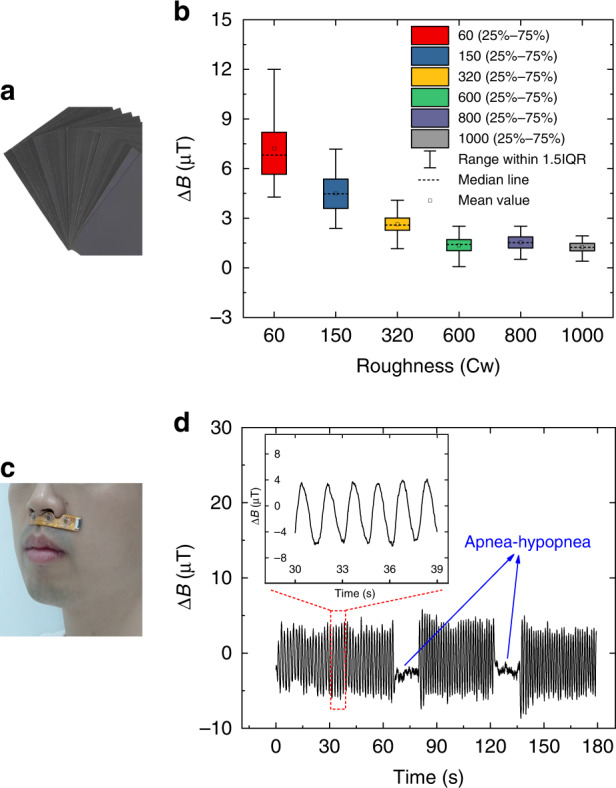
Applications of the sensor. **a** Abrasive paper with different roughnesses. **b** Results of characterizing different surface roughnesses. **c** Test scenario of sleep apnea monitoring. **d** Test results of sleep apnea monitoring. Inset: enlarged view of the normal respiratory waveform

Surface roughness characterization is essential in many areas, such as mechanical manufacturing, object identification and food status classification^26^. Benefiting from the excellent performance in micro force detection, this sensor can be applied to characterize roughness. Several sheets of abrasive paper with different roughnesses were used as the characterization object, as shown in Fig. 5a. The roughness of the selected abrasive paper was 60 Cw, 150 Cw, 320 Cw, 600 Cw, 800 Cw, and 1000 Cw (where Cw is the unit of roughness). As the value increases, the roughness decreases. The size, spacing and average height of the particles on each abrasive paper were different. The abrasive paper was attached to a flat test platform. Then, the sensor was fixed upside down on a linear guide, and the cilium was kept in contact with the abrasive paper. The guide was moved horizontally to make the cilium slide across the abrasive paper. The resulting data are plotted as a box graph without outliers in Fig. 5b. The IQR (interquartile range) is defined as the distribution range of 25–75% of the data after sorting, which is shown in Fig. 5b as the height of the colorful boxes. The distribution of the data becomes more concentrated as the roughness decreases (the size and spacing of particles are reduced). The mean and median values also tend to decrease. This is because the decrease in the average height of the particles leads to a smaller bending angle of the cilium.

### Sleep apnea monitoring

In addition to sensing micro forces, the ability of our sensor to achieve airflow perception also has great application potential in intelligent robots and medical treatment. We successfully applied this tactile sensor to the monitoring of sleep apnea. Sleep apnea hypopnea syndrome (SAHS) is a common sleep disorder. Apnea and dyspnea occur repeatedly during sleep, resulting in insufficient oxygen supply to patients. It may induce chronic diseases such as hypertension and coronary heart disease^27^. It is necessary to detect SAHS through a sleep respiratory monitoring system. When collecting respiratory signals, conventional devices such as polysomnography require an oxygen tube to be inserted deeply into the patient’s nostrils, which causes great discomfort. We have already shown that our sensor is sensitive enough to detect airflow velocity in Fig. 4f. Therefore, the sensor can be made into a wearable “artificial beard” to conveniently monitor respiratory signals. A simulation experiment was carried out to verify the effect. The test scenario is shown in Fig. 5c. A volunteer simulated two apneas during a three-minute breathing period. The results are shown in Fig. 5d. Apneas occurred at the 65th and 125th seconds, and the output waveform of the sensor was significantly different from that of normal breathing. The time, duration and number of sleep apnea events can be obtained through real-time procedural processing. Therefore, the sensor designed in this study can serve as a convenient device for pathological analysis. Compared with polysomnography, the sensor has the advantages of small size, low cost and high comfort, and it is more suitable for household use.

## Conclusions

A double-layer magnetic cilia sensor was designed in this work, which could detect both force and airflow motion. The double-layer structure could significantly improve the magnetic particle content (over 70%) while ensuring the flexibility of cilia. The MTP method was further proposed to produce cilia with larger aspect ratios (over 30:1). The resolution of the sensor to micro forces is 2.1 µN, while the sensitivity is 0.63 µT/µN. This performance is superior to other magnetic tactile sensors reported previously. The sensor also shows excellent detection ability for airflow velocity, with a sensitivity of 1.43 µT/(m/s). A repeated bending experiment has demonstrated the durability of the sensor, and the use of a reference sensor can reduce the interference of the external magnetic field. Therefore, the sensor is suitable in wearable electronics. Finally, the proposed sensor shows great performance in surface roughness characterization and sleep apnea monitoring, which shows that the sensor has great application potential in intelligent robots and medical treatment.

### Supplementary information

Supplemental Material
