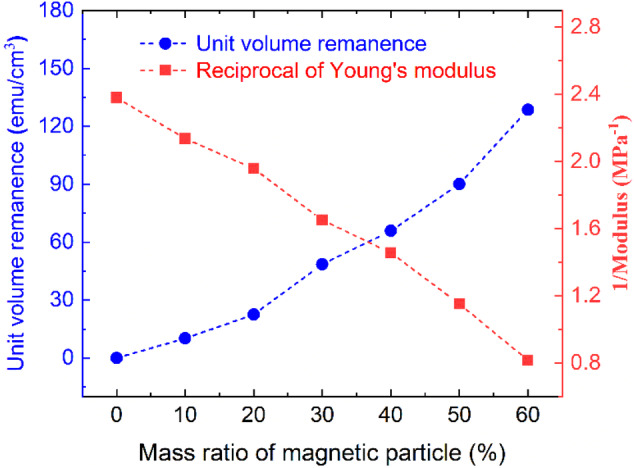# Correction: A tactile and airflow motion sensor based on flexible double-layer magnetic cilia

**DOI:** 10.1038/s41378-024-00710-8

**Published:** 2024-06-24

**Authors:** Jiandong Man, Junjie Zhang, Guangyuan Chen, Ning Xue, Jiamin Chen

**Affiliations:** 1 State Key Laboratory of Transducer Technology, Aerospace Information Research Institute, Chinese Academy of Sciences, 100190 Beijing, People’s Republic of China; 2 School of Electronic, Electrical and Communication Engineering, University of Chinese Academy of Sciences, 100049 Beijing, People’s Republic of China

**Keywords:** Electrical and electronic engineering, Nanoparticles

Correction to: *Microsystems & Nanoengineering*

10.1038/s41378-022-00478-9 published online 17 January 2023

After the publication of this article^1^, we noticed one necessary correction as follows:

**Correction 1:**

Figure 4a needs to be replaced with the new image shown below.