# Supplementary material for: A tactile and airflow motion sensor based on flexible double-layer magnetic cilia
Source: Microsyst Nanoeng. 2023 Jan 17;9:12. doi: 10.1038/s41378-022-00478-9 (PMC9845383; doi:10.1038/s41378-022-00478-9)
Supplement: Supplementary file 1 — Supplemental Material [file 41378_2022_478_MOESM1_ESM.docx]

**Supplementary Materials**

# A tactile and airflow motion sensor based on flexible double-layer magnetic cilia

*Jiandong Man^1,2^, Junjie Zhang^1,2^, Guangyuan Chen^1^, Ning Xue^1,2^ and Jiamin Chen^1,2,*^*

^1^State Key Laboratory of Transducer Technology, Aerospace Information Research Institute, Chinese Academy of Sciences, Beijing 100190, People’s Republic of China

^2^School of Electronic, Electrical and Communication Engineering, University of Chinese Academy of Sciences; Beijing 100049, People’s Republic of China

^*^Correspondence: Jiamin Chen ([chenjm@aircas.ac.cn](mailto:chenjm@aircas.ac.cn))

**Fig. S1 Influence of curing temperatures on Young's modulus of PDMS.**

**Fig. S2 Response of placing and then removing a feather (18 mg) slowly on the sensor.**
